# A moderate static magnetic field promotes *C. elegans* longevity through cytochrome P450s

**DOI:** 10.1038/s41598-022-20647-0

**Published:** 2022-09-27

**Authors:** Mengjiao Song, Shiming Dong, Xiangfei Zhang, Yumin Dai, Xin Zhang, Yidong Shen

**Affiliations:** 1 State Key Laboratory of Cell Biology, Innovation Center for Cell Signaling Network, CAS Center for Excellence in Molecular Cell Science, Shanghai Institute of Biochemistry and Cell Biology, University of Chinese Academy of Sciences, Chinese Academy of Sciences, 320 Yueyang Rd., Shanghai, 200031 China; 2 High Magnetic Field Laboratory, Hefei Institutes of Physical Science, Chinese Academy of Sciences, Hefei, China; 3 Science Island Branch of Graduate School, University of Science and Technology of China, Hefei, China

**Keywords:** Genetics, Ageing

## Abstract

Ageing is co-regulated by genetic and environmental factors. Life on earth lives and evolves in a mild geomagnetic field. Yet, the biological effects of a moderate magnetic field on ageing and the underlying genetic mechanisms remain barely unknown. Here, we report that a moderate static magnetic field (SMF) extends the lifespan of *Caenorhabditis elegans*, a well-established model organism in ageing research. Consistently, the SMF-treated worms show improved motility and mitochondrial function when aged. We identified from the transcriptomic changes upon SMF treatment that the upregulation of three cytochrome P450 genes are required for SMF-induced longevity. Our findings thus reveal that proper SMF treatment could promote longevity through the well-conserved cytochrome P450 enzymes.

## Introduction

Ageing is regulated by intrinsic genetic pathways and in response to extrinsic environmental cues^1,2^. Diet and temperature are widely reported to modulate longevity through a network of molecular signalling^1,2^. The magnetic field is another critical environmental factor to all life on earth, which has a mild dipolar geomagnetic field (GMF) of 25–65 μT. Whereas artificial magnetic fields of high energy are hazardous by ionizing and thermal effects^3^, GMF is known to have many significant biological effects. Magnetotactic bacteria sense GMF for cellular migration^4^. Other organisms across taxa, such as butterflies, salmon, and birds, are considered to navigate over long distances by tracing GMF^5–7^. The moderate magnetic field has recently been shown to regulate immune cell function and redox homeostasis^8–10^. Given its various biological effects, it is intriguing to explore the potential influence of the moderate magnetic field in ageing and the underlying mechanisms.

The nematode *Caenorhabditis elegans* is a well-established model organism in ageing research, with conserved ageing phenotypes and mechanisms^11^. Adult *C. elegans* is around 1 mm in length and 31–72 μm in diameter. In lab, these tiny worms are grown on a two-dimensional bacteria lawn^12^. These features make it easy to treat multiple worms and different worm tissues with similar magnetic field intensity. Moreover, *C. elegans* was suggested to sense GMF during vertical burrowing migrations^13^. Here, we investigated the effect of a moderate magnetic field on worm ageing. Our results indicate that a static magnetic field (SMF) of 10 mT extends worms lifespan and enhances the motility of aged worms, potentially through inhibiting the ageing-related changes of mitochondrial morphology and function. We further found that SMF treatment upregulates a group of cytochrome P450 genes to induce longevity. Our findings thus reveal the biological effect of SMF on ageing and underscore the role of cytochrome P450s in it.

## Results

### Moderate SMF promotes the longevity of *C. elegans*

**Figure 1:**
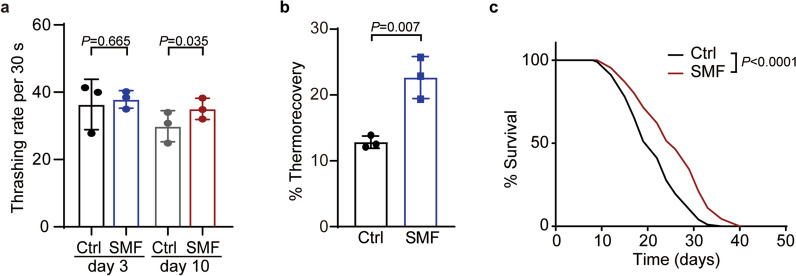
A moderate static magnetic field promotes worms longevity. (**a**) Worms treated with SMF of 10 mT were examined for their thrashing rates at indicated ages. Paired *t*-test. (**b**) SMF of 10 mT increases the recovery rate from heat shock in worms at day 1 of adulthood. Unpaired *t*-test. (**c**) SMF of 10 mT extends the lifespan of WT worms. Mantel-Cox Log Rank test. See Supplementary Table 1 for details.

SMFs stronger than 200 mT were reported to accelerate development and promote ageing^14^. To explore the potential effects of moderate SMFs on longevity, we therefore prepared three different permanent magnetic plates below 200 mT. At the position of worm culture, which is 5 mm over the plate, the plates generate SMFs of around 10 mT, 50 mT, and 100 mT, respectively (Supplementary Fig. S1a). Wild type (WT) worms were grown in these SMFs and scored for health conditions by their motilities at day 3 and 10 of adulthood (D3 and D10)^15^. After entering adulthood, lab-cultured worms have an average reproductive period of ~ 7 days and a lifespan of ~ 20 days^11^. Therefore, the worms at D3 and D10 are considered respectively as young and aged in our assays. At D3, the SMF of 50 mT exhibited a marginal effect by increasing worms’ motility by 3.7%, whereas the other two showed no influences (Fig. 1a, Supplementary Fig. S1b). The worms’ motility at D10 was not changed upon the exposure to the SMFs of 50 mT or 100 mT (Supplementary Fig. S1b). In contrast, the SMF of 10 mT significantly enhanced worms’ motility by 21.5% at D10 (Fig. 1a, Supplementary Fig. S1b), indicating that a proper treatment of moderate SMF improves the health of aged worms. Stress resistance in young adults is tightly related to ageing^16^. We then examined the heat resistance of 10 mT SMF-treated worms at D1. 22.6% SMF-treated worms recovered from a 5.5 h heat shock at 35 °C, whereas the thermorecovery rate of untreated worms was 12.8% (Fig. 1b). Consistent with its positive effect on healthspan and heat resistance, the exposure to 10 mT SMF also extended the median lifespan by 18% and the maximum lifespan by 11% (Fig. 1c and Supplementary Table 1). Taken together, SMF of 10 mT promotes the longevity of worms.

### Moderate SMF alters the morphology and function of mitochondria

**Figure 2:**
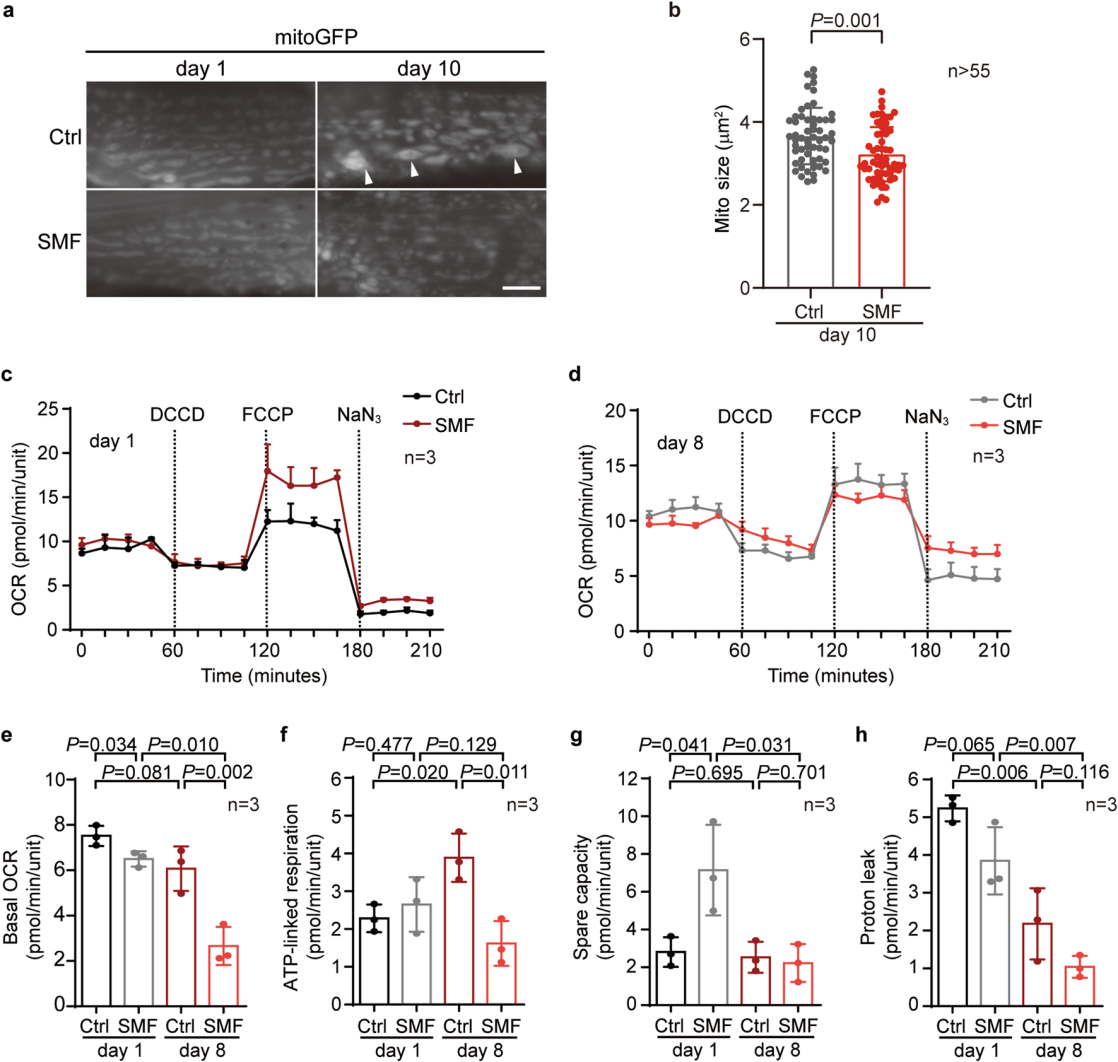
Mitochondria is under the regulation of the static magnetic field. (**a**) SMF of 10 mT regulates the ageing-induced change of mitochondrial morphology. Mitochondria in body wall muscle were examined at indicated ages. Arrowheads denote representative swollen mitochondria. Scale Bar: 2 μm. (**b**) SMF reduces the average sizes of mitochondria in body wall muscle at day 10 of adulthood. Unpaired *t*-test. (**c**) and (**d**) Seahorse Mito Stress Test in worms treated with SMF at indicated ages. (**e**)–(**h**). Four benchmarks of mitochondrial function, namely basal OCR (**e**), ATP-linked respiration (**f**), spare capacity (**g**), and proton leak (**h**) in Ctrl or SMF-treated worms at indicated ages. Unpaired *t*-test.

A recent report shows that moderate SMF promotes mitochondrial respiration^8^. Since mitochondria are also critical in ageing^1^, we next checked the impact of SMF on mitochondria morphology in the body wall muscle (BWM) of worms at different ages. As reported^17,18^, mitochondria were in a tubular and well-organised network in young worms at day 1 of adulthood (D1), whereas they appeared to be swollen and fragmented in aged worms at D10 (Fig. 2a and b). 10 mT SMF treatment did not affect mitochondria morphology in D1 worms but suppressed the age-related changes in the mitochondria network (Fig. 2a and b).

The morphology of mitochondria is tightly linked to its function. We then used Seahorse Mito Stress Test to measure mitochondria function in D1 and D8 worms by four benchmarks: basal OCR, ATP-linked respiration, spare capacity, and proton leak (Fig. 2c and d). The four mitochondrial benchmarks respectively show the mitochondrial function under baseline conditions, its contribution to the cellular energy needs, its flexibility to respond to energetic demand, and its damage^19^. In agreement with its effect on mitochondria morphology, SMF of 10 mT significantly reduced basal OCR, ATP-linked respiration, and proton leak in aged worms (Fig. 2e–h). Moreover, although SMF did not affect mitochondria morphology in the BWM of D1 worms (Fig. 2a and b), it mildly suppressed basal OCR and remarkably increased spare capacity at D1 (Fig. 2e and g). Proton leak was also slightly decreased upon SMF treatment at D1, albeit without statistical significance (*P* = 0.065) (Fig. 2h). Therefore, moderate SMF controls mitochondria in both young and aged worms.

Mitophagy is critical to the morphology and function of mitochondria and in turn to ageing^20,21^. The treatment of 10 mT SMF did not change the induction of mitophagy at D10 (Supplementary Fig. S2), suggesting that mitophagy may not be involved in the SMF-induced regulation on mitochondria.

### Moderate SMF modulates the expression of cytochrome P450s

**Figure 3:**
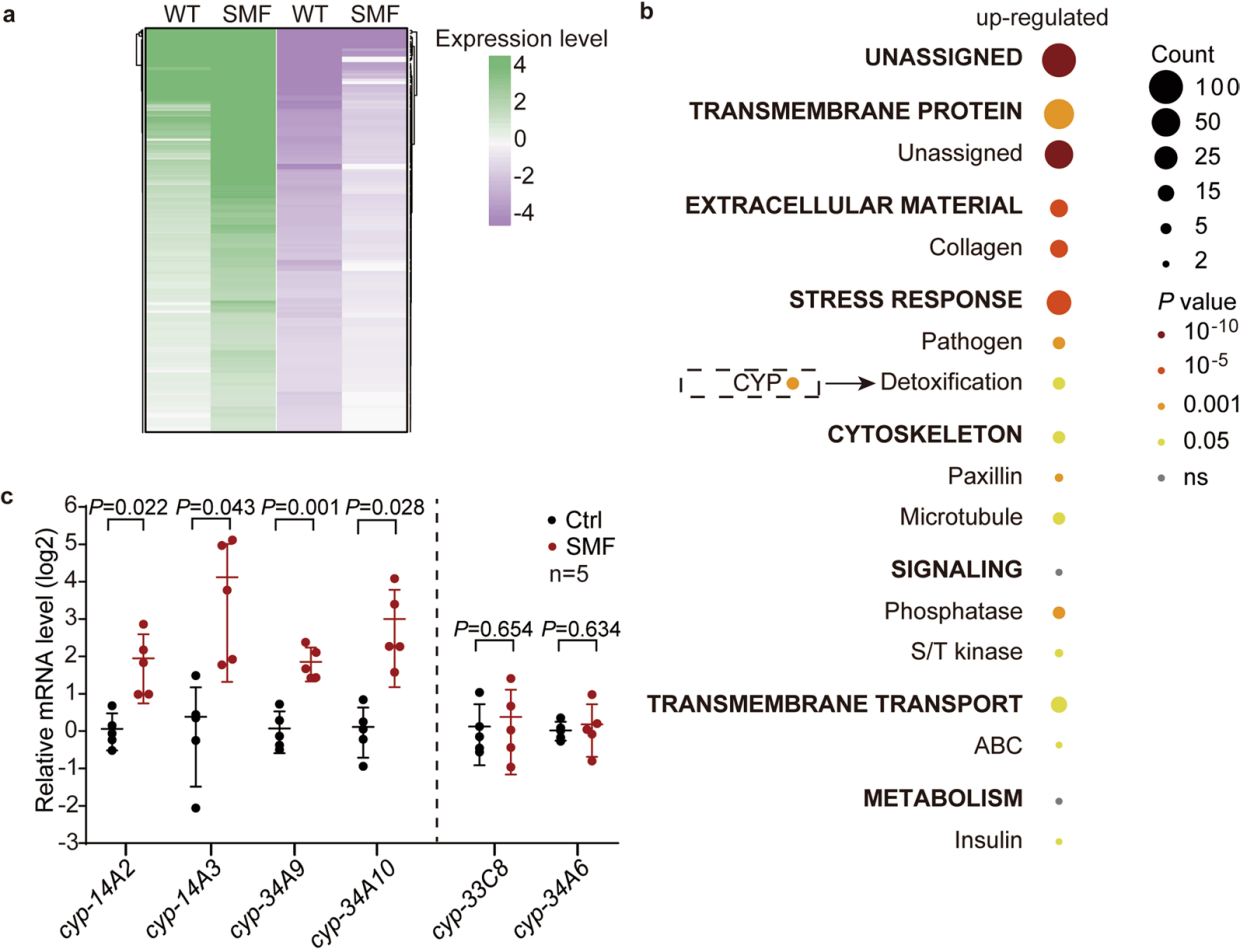
A group of cytochrome P450 genes are induced by the static magnetic field. (**a**) A heatmap depicting the differentially expressed genes (DEGs) in worms at day 0 of adulthood upon 10 mT SMF treatment. (**b**) Gene enrichment analysis of the upregulated DEGs upon SMF treatment by WormCat. Categories 1 and 2 are differentiated by capitalization and bold fonts. Category 2 are shown below the corresponding Category 1. Categories 1 with RGS bigger than 10 were shown. See Supplementary Table 3 for more details. (**c**) RT-qPCR analysis of the indicated CYP genes in day 1 adult worms with or without SMF treatment. Unpaired *t*-test.

To explore how SMF prolongs worm longevity and alters mitochondria activity (Figs. 1 and 2), we first examined AMPK, a pivotal metabolic regulator controlling mitochondria^17,18,22^. AMPK activation can be monitored by phosphorylation at its conserved Thr172^23^. In contrast to our hypothesis, SMF of 10 mT did not change the level of p-AMPK in worms at D1 (Supplementary Fig. S3), implying that AMPK may not be involved in SMF-induced longevity. For clues of the molecular mechanism underlying SMF treatment, we next profiled the 10 mT SMF-induced transcriptomic changes in freshly moulted adults (D0). 826 and 494 genes were respectively up- and down-regulated by SMF with a change bigger than twofold (Fig. 3a and Supplementary Table 2). Analysis using WormCat indicates that the upregulated genes are enriched in stress responses (Fig. 3b and Supplementary Table 3), which are critical to longevity^24,25^. Consistent with its effect on mitophagy (Supplementary Fig. S2), 10 mT SMF did not change the expression of *pink-1*, *pdr-1*, and *dct-1*, three critical mitophagy genes, by either RNA-Seq or RT-qPCR results (Supplementary Fig. S4 and Supplementary Table 2)^20^. Instead, a group of cytochrome P450 (CYP) genes aroused our interest among the upregulated genes regulating stress resistance. The CYP genes are closely related to mitochondria function^26^. RT-qPCR results confirmed the upregulation of *cyp-14A2*, *cyp-14A3*, *cyp-34A9*, and *cyp-34A10* (Fig. 3c).

oPOSSUM-3 predicted *lin-14* as the transcription factor driving the CYP genes, based on the over-represented conserved transcription factor binding sites in their promoters^27^. However, mutating *lin-14* did not block the SMF-induced upregulation of CYP genes (Supplementary Fig. S5). Worms were reported to sense GMF through *tax-4* in neurons^13^. We then examined whether SMF increased the expression of CYP genes through *tax-4*. Whereas mutating *tax-4* did not affect the upregulation of *cyp-14A3* upon SMF treatment, it could suppress the SMF-induced increase of *cyp-14A2*, *cyp-34A9*, and *cyp-34A10* (Supplementary Fig. S5), implying that neuronal network could be involved in the response to SMF.

### The upregulation of cytochrome P450s are required for SMF-induced longevity

**Figure 4:**
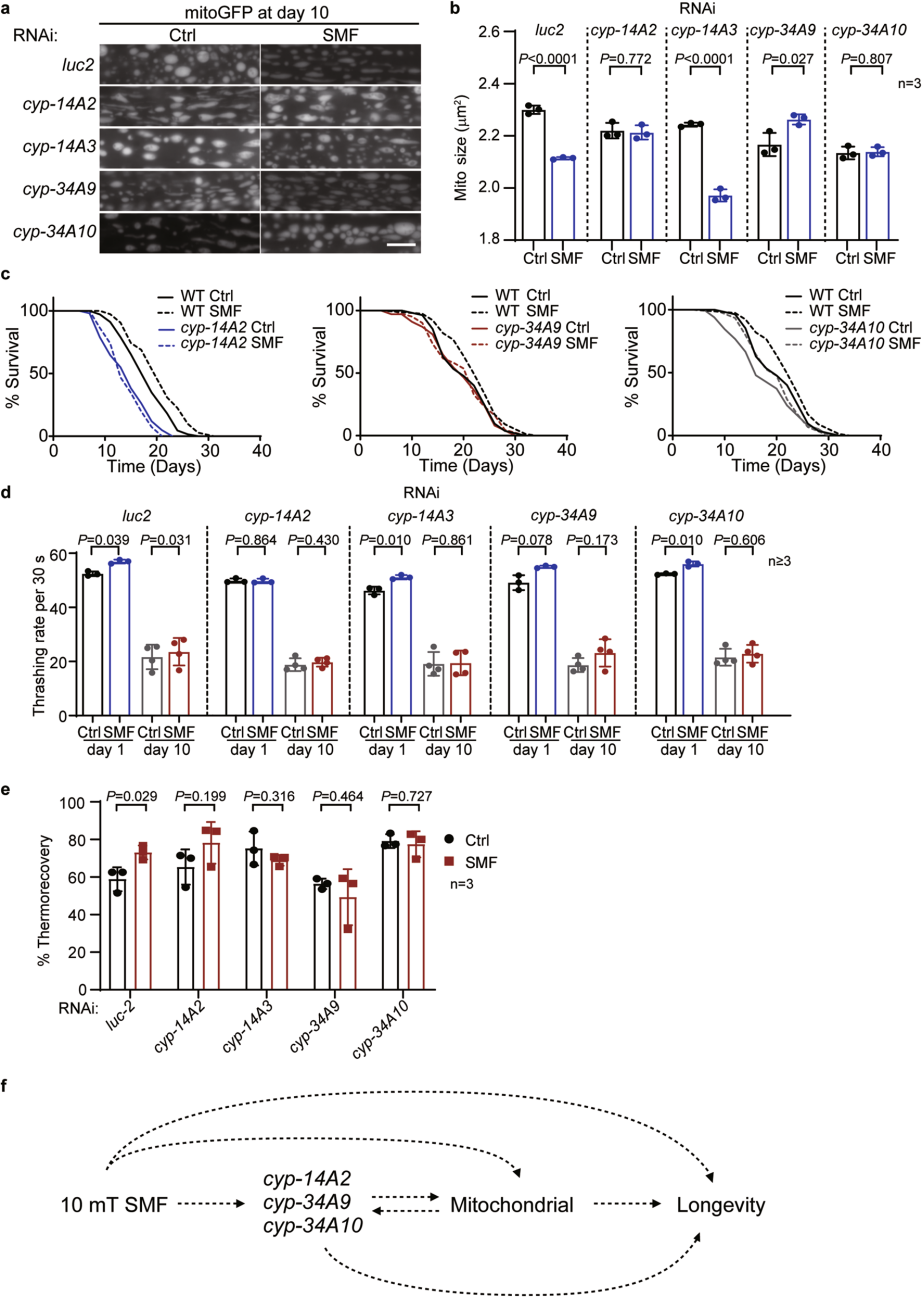
Cytochrome P450s are required for SMF-induced longevity. (**a**) Worms were treated with indicated RNAi and grown with or without SMF till day 10 of adulthood. Mitochondrial morphology in the body wall muscle was then examined. Scale bar: 5 μm. (**b**) RNAi against *cyp-14A2*, *cyp-34A9*, or *cyp-34A10* blocks the effect of SMF on mitochondrial size at day 10 of adulthood. Unpaired *t*-test. (**c**) The survival curves of worms under indicated treatments. (**d**) The thrashing rates of worms at day 1 and day 10 of adulthood under indicated treatments. Paired *t*-test. (**e**) RNAi against the indicated CYP genes suppresses the improved thermorecovery by SMF treatment at day 1 of adulthood. Note that the worms under RNAi were fed with *E. coli* HT115, whereas the worms in Fig. 1b with *E. coli* OP50. Although different food caused different response to heat shock, SMF consistently increased thermorecovery. (**f**) A schematic summary of the SMF-induced longevity in *C. elegans*.

We then explored the role of CYPs in SMF-induced longevity. Since CYPs are involved in proper mitochondria function^26^, we first checked the SMF-induced changes of mitochondria morphology. Under the control treatment of *luc2* RNAi, SMF of 10 mT suppressed the deterioration of the mitochondria network (Fig. 4a and b), as we observed (Fig. 2a and b). RNAi against *cyp-14A2* or *cyp-34A10* abrogated the effect of 10 mT SMF on mitochondria morphology, whereas inhibiting *cyp-14A3* showed no impact, and *cyp-34A9* RNAi further disrupted the mitochondria network (Fig. 4a and b). We then focused on *cyp-14A2*, *cyp-34A9*, and *cyp-34A10*, which block SMF-induced changes of mitochondria network, and examined their functions in longevity. The lifespans of these three CYPs knockout mutants were unchanged by 10 mT SMF (Fig. 4c and Supplementary Table 1). Moreover, when these CYPs were suppressed by RNAi, SMF of 10 mT no longer increased worms thrashing rates at D10 (Fig. 4d). The improved heat resistance upon SMF treatment was consistently abrogated by RNAi against the CYPs (Fig. 4e). Therefore, the three CYPs are essential to SMF-induced longevity.

## Discussion

In this study, we used the nematode *C. elegans* as a model to explore the biological effect of static magnetic field (SMF) on ageing. Our data indicate that a moderate static magnetic field of around 10 mT induce significant transcriptomic changes in worms, alleviates the deterioration of mitochondria in ageing, and extends both the health span and life span of worms. A group of cytochrome P450 (CYP) genes play a critical role in SMF-induced longevity (Fig. 4f).

Environmental cue is critical to ageing. Whereas food, temperature, and light have been extensively studied in the biology of ageing^28,29^, the role of the magnetic field remains unknown. All organisms on earth de facto live and evolve in the geomagnetic field (GMF). Magnetic fields have also been reported to have various biological effects^3,5^. It is natural that ageing, one of the most important genetic traits, could also be regulated by the magnetic field. Intervention of the magnetic field thus could alter the signalling network evolved in adaptation to GMF and modulate ageing. Indeed, we found that a moderate SMF of 10 mT promotes a series of longevity phenotypes, including motility, heat resistance, and lifespan (Fig. 1). Similar with a previous report^14^, we did not observe obvious defects, such as body size and brood size, in 10 mT SMF-treated adult worms. Nevertheless, we can not exclude that the SMF of 10 mT could cause some subtle negative effects on worms.

The SMF of 10 mT is several hundred times stronger than GMF. Therefore, its effect on ageing does not necessary mean that GMF could promote longevity but may rather be from an artificial modulation of the signalling pathways naturally responsive to GMF. Nevertheless, it will still be interesting to check animal ageing in the magnetic shield condition to figure out the role of GMF in longevity. Meanwhile, SMF of 10 mT is common in outer space, suggesting that the extreme conditions in space may interact with the molecular network evolved on earth and produce unexpected biological effects. Testing organismal ageing in space is definitely of great interest.

As in the case of other environmental factors, a proper dosage of SMF treatment is crucial to its anti-ageing effect. SMF of 200 mT was shown to shorten the lifespan of worms^14^. Among the three SMFs in our test, only the SMF of 10 mT affects ageing (Fig. 1 and Supplementary Fig. S1). Similarly, a recent report showed that 0.3 T SMF is more potent than 0.6 T SMF in inducing cytokine secretion of CD8^+^ T cells^8^. The dose-dependent feature of SMF-induced biological effects makes it intriguing to find optimal SMF treatment in the future. Due to technical limitation, we were unable to test SMFs below 10 mT in this study. It will be interesting to check animals ageing in lower SMFs (e.g., 1 and 5 mT). It also raises attention for future studies because it is technically challenging to make an SMF with even intensity in a three-dimensional (3D) space. Therefore, *C. elegans* could be an excellent model in studying the SMF-induced longevity effect. In addition to its advantages in ageing research, lab-cultured worms on plates can be viewed as living in a 2D manner, facilitating the adjustment and maintenance of its SMF treatment. When it comes to other organisms such as mice, the spatial distribution of SMF should be considered when interpreting the biological effect of SMF.

As with other ageing interventions, SMF of 10 mT induces a remarkable transcriptomic change. In addition to the CYP genes highlighted in this study, the differentially expressed genes upon SMF treatment are enriched in multiple gene sets related to ageing. For example, E3 ligases and innate immunity genes, which are essential in protein homeostasis and pathogen defence^30,31^, were also upregulated by SMF (Supplementary Table 3). Therefore, SMF could induce longevity through a complex genetic signalling network.

In the SMF-regulated genes, our data show that three CYPs, *cyp-14A2*, *cyp-34A9* and *cyp-34A10*, are upregulated by SMF and essential to SMF-induced longevity (Figs. 3 and 4). Blocking their function via either genetic mutation or RNAi completely abolishes the anti-ageing phenotypes upon SMF treatment, including mitochondrial morphological changes, improved heat resistance, extended healthspan and lifespan (Fig. 4). CYPs are well known in drug metabolism^32,33^. *cyp-34A9* and *cyp-34A10* were shown to catalyse tolbutamide metabolism^34^. More importantly, CYPs are important enzymes in the oxidation of endogenous compounds such as lipids^35^. *cyp-14A2* was reported to metabolise eicosapentaenoic acid (EPA), the predominant polyunsaturated fatty acid of *C. elegans*^36^. Therefore, the three SMF-upregulated CYPs could promote longevity through detoxification and lipid metabolism.

CYPs have intensive interaction with mitochondria. Mitochondria is a major cellular organelle of CYPs localisation^26^. The mitochondrial electron transport system serves as an electron donor for mitochondrial CYPs catalytic activity, whereas the products of CYP-dependent metabolism affect mitochondrial functions^26,35^. CYP2U1, the mammalian ortholog of the three CYPs in SMF-induced longevity, is localised in mitochondria and controls mitochondrial morphology^37^. As the three CYPs also regulate the morphological changes of mitochondria during worm ageing (Fig. 4), it will be interesting to clarify their interaction with mitochondria further and to pursue the potential role of CYP2U1 in mammal ageing.

In summary, this study reveals the role of SMF in worm ageing and identifies a set of CYPs as essential genes for SMF-induced longevity. However, it remains a puzzle how SMF triggers this biological effect. Studies in animal navigation show that some animals, such as birds and butterflies, can sense magnetic fields^5–7^. *C. elegans* was also proposed to have magneto sensitive neurons for geomagnetic orientation^13^, implying that SMF might regulate worm ageing through these neurons. Consistently, we found that mutating *tax-4* could affect the SMF-dependent upregulation of three CYP genes. Moreover, recent studies on cellular effects of SMFs, together with our findings, highlight the role of mitochondria^8,9^, suggesting mitochondria could be an SMF sensor in the cell. Exploring the transcription factors driving the transcriptomic changes upon SMF treatment is another exciting issue to pursue. It is intriguing and essential to study how worms sense SMF alteration and turn on longevity response in the future.

## Methods

### Worm culture and RNAi interference

*Caenorhabditis* *elegans* were grown with standard techniques on NGM agar plates seeded OP50 at 20 °C, unless otherwise noted^38^. The Worm strains used in this study are listed in Supplementary Table 4. Some strains were provided by the CGC, which is funded by NIH Office of Research Infrastructure Programs (P40 OD010440). For synchronization, eggs laid in the desired time window (4 h to O/N) were collected unless otherwise noted.

RNAi experiments were performed as described^39^.

### Permanent magnetic plates

N35 AlNiCo permanent magnetic plates were from the Hangzhou Permanent Magnet Group Co., Ltd. The size of each plate is 25 cm × 25 cm. Their thickness are 6.5 mm, 12.5 mm, and 25 mm to generate SMFs of around 10 mT, 50 mT, and 100 mT at 5 mm over the plate, respectively. The magnetic field strength was measured by KANETEC TESLA METER MODEL TM-801.

### Lifespan assays

Adult lifespan analysis was performed as previously reported. In brief, synchronized adult worms were transferred onto fresh plates every other day during the reproductive period. Worm survival was scored every 2–3 days. Worms not responding to prodding were considered as dead. Worms undergoing internal hatching, bursting vulva, or crawling off the plates were censored. Statistical analysis was performed with the Mantel-Cox Log Rank method.

### Plasmid construction

To generate plasmids for RNAi, 1479 bp of *cyp-14A2* cDNA, 1497 bp of *cyp-14A3* cDNA, 1551 bp of *cyp-34A9* cDNA, and 1500 bp of *cyp-34A10* cDNA was PCR-amplified from N2 cDNA and cloned into T444T^40^. For the control of RNAi assays, 1675 bp of *luc2* cDNA was PCR amplified from *L4440::luc2* and cloned into T444T. *L4440::luc2* was a gift from Antebi lab (MPI-AGE). The sequences of primers used are listed in Supplementary Table 5.

### Microscopy

For mitochondrial image analysis, worms were anaesthetized using 5 mM levamisole and mounted on 5% agar pads on glass slides under coverslips. Fluorescence images were acquired with an Olympus BX53 microscope, captured at 60 × magnification. The mitochondrial size was measured using Imaris (Oxford Instruments). For mitophagy analysis, worms were imaged on a Zeiss LSM880 Airyscan microscope.

### Motility

For thrashing rates, worms at indicated ages were transferred into a 96-well plate, with each well supplemented with 150 μl of M9. A Nikon D4 camera mounted on an Olympus SZX16 stereomicroscope was used for video recording. The thrashing rate was subsequently scored from videos. 70–90 animals from at least three independent experiments were examined for each genotype or treatment.

### Heat resistance assay

To assess worms heat resistance, thermorecovery was measured as reported^41^. In brief, worms were heat shocked at 35 °C for 5.5 h and then grown at 20 °C for 24 h to recover. Worms able to crawl away without abnormal, jerky movement or paralysis upon prodding were classified as ‘recovered’. Fewer than 30 worms were on each plate during the assay.

### Seahorse assay

As reported, oxygen consumption was measured using a Seahorse XFe24 Analyzer (Agilent Technologies)^42^. In brief, worms were washed off bacteria and transferred into a 24 well plate (20–40 worms/well). Basal respiration was first measured 8 times. 20 μM DCCD (Sigma-Aldrich, Cat# 379115), 25 μM FCCP (Sigma-Aldrich, Cat# C2920), and 10 mM NaN_3_ were sequentially added. Following the addition of each drug, ATP-linked respiration, maximal respiration, and azide response were measured for 8 times, 8 times, and 4 times, respectively. Oxygen consumption rates were normalised to the number of worms in each well. 3–6 technical replicates were included in each biological replicate.

### RNA preparation and RT-qPCR

Synchronized worms were harvested at indicated ages in TRIzol (Invitrogen, Cat# 15596018). After adding 0.2 volume of chloroform and centrifugation at 12,000 g for 15 min at 4 °C, the top aqueous phase was collected and subjected to total RNA preparation using RNeasy Mini kit (QIAGEN, Cat# 74104) with on-column DNase I (QIAGEN, Cat# 74106) digestion.

For RT-qPCR, cDNA was subsequently generated by iScript™ Reverse Transcription Supermix for qPCR (Bio-Rad, Cat# 1708841). qPCR was performed using Bestar® Sybr Green qPCR Master Mix (DBI Bioscience, Cat# DBI-2043) on a QuantStudio™ 6 Flex Real-time PCR System (Applied Biosystems) or a CFX384 Touch™ Real-Time PCR Detection System (Bio-Rad). Four technical replicates were performed in each reaction.

### RNA-Seq and data analysis

RNA libraries were prepared by BGI and sequenced on a BGISEQ-500 sequencer. Raw sequencing reads were cleaned by removing adaptor sequences, reads with poly-N sequences, and low-quality reads. Approximately 21.6 million clean reads were mapped to the *C. elegans* reference genome (WBcel235) using HISAT/Bowtie2 tools. Normalisation was performed after data were mapped, and then FPKM (fragments per kilobase per million mapped reads) was calculated using RESM software. Genes with an FPKM bigger than 0.15 were considered as detected. The genes up- or down-regulated more than two-fold upon SMF treatment were considered differentially expressed. Gene set enrichment was analysed using WormCat by default settings^24^. Significance scores were reported as Fisher’s exact test *P* values. Terms were considered significant if the WormCat reported *P*-value score was smaller than 0.05.

### Western blotting

Synchronized worms were grown to day 1 of adulthood and harvested in 4 × SDS gel-loading buffer (Takara, Cat# 9173). After three rounds of freeze and thaw, worms were further lysed by incubation at 95 °C for 5 min. Proteins were separated by reducing SDS-PAGE and transferred to PVDF membranes. Membranes were blotted with antibodies against p-AMPK (CST, Cat# 4188 s, dilution: 1:1000) and α-tubulin (Sigma-Aldrich, Cat# T5168, dilution: 1:2000). Anti‐mouse secondary antibody conjugated with horseradish peroxidase (Life Technologies, Cat# G21040, 1:5000) was used to detect anti‐α‐tubulin, and anti‐rabbit secondary antibody (Life Technologies, Cat# G21234, 1:5000) was used for detecting anti‐p‐AMPK primary antibodies. Signals of western blotting were captured by a Tanon™ 5200 Chemiluminescent Imaging System and measured using Adobe Photoshop. Background signals were subtracted as reported^43^.

### Statistical analysis

Results are presented as Mean ± SD unless otherwise noted. Statistical tests were performed as indicated using GraphPad Prism (GraphPad software). Detailed statistics information was shown in Supplementary Table 1.

## Supplementary Information

Supplementary Figure S1.

Supplementary Figure S2.

Supplementary Figure S3.

Supplementary Figure S4.

Supplementary Figure S5.

Supplementary Figure S6.

Supplementary Legends.

Supplementary Table 1.

Supplementary Table 2.

Supplementary Table 3.

Supplementary Table 4.

Supplementary Table 5.

## Data Availability

The RNA-Seq data from this publication have been deposited to the SRA database [https://www.ncbi.nlm.nih.gov/sra] and assigned the reviewer links as below: https://dataview.ncbi.nlm.nih.gov/object/PRJNA793996?reviewer=ljpgregktvtouealqqbe4nm75o.
