# Supplementary figures and images for: A moderate static magnetic field promotes C. elegans longevity through cytochrome P450s
Source: Sci Rep. 2022 Sep 27;12:16108. doi: 10.1038/s41598-022-20647-0 (PMC9515093; doi:10.1038/s41598-022-20647-0)

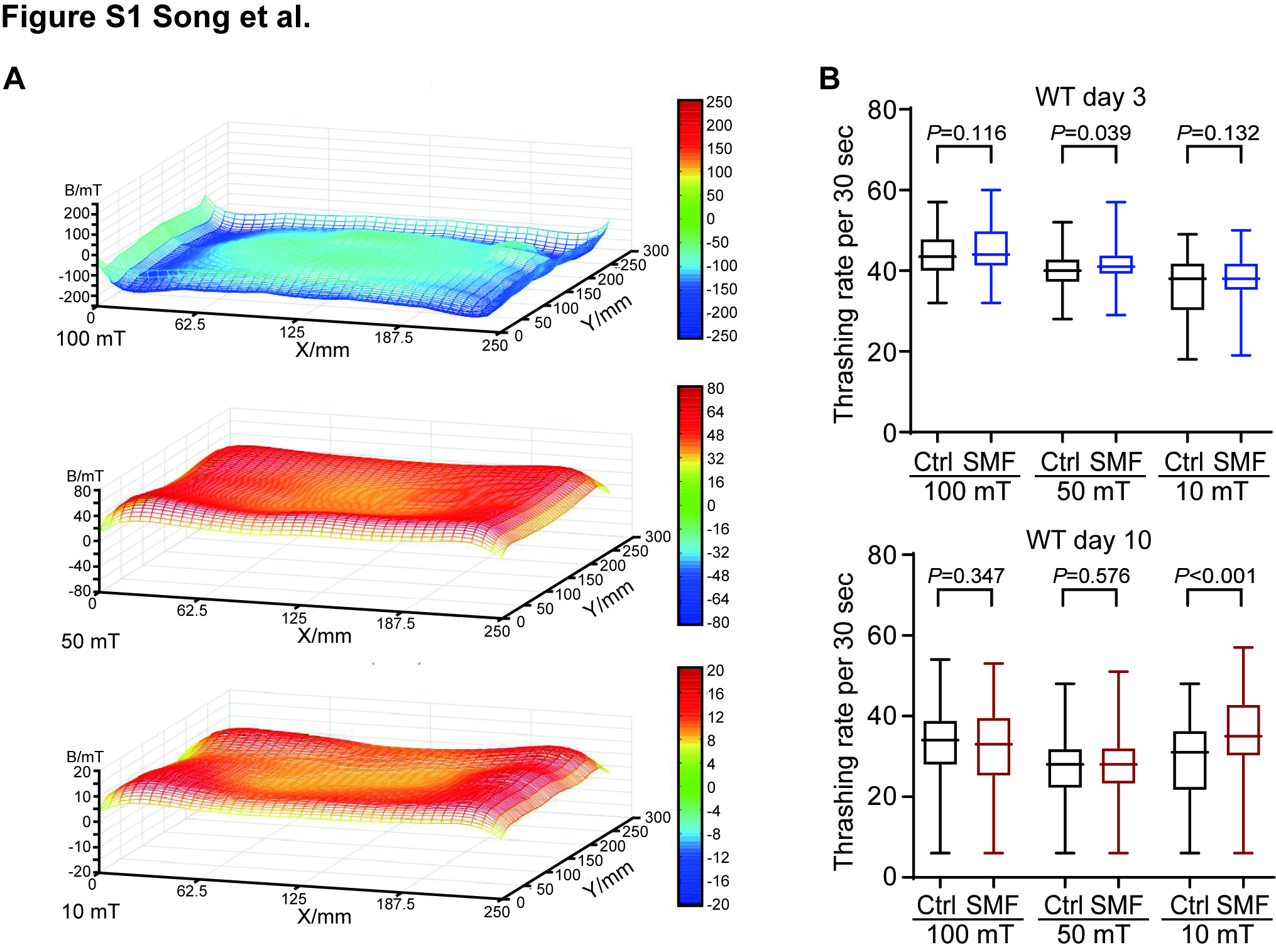

Supplement: Supplementary file 1 — Supplementary Figure S1. [file 41598_2022_20647_MOESM1_ESM.tif]

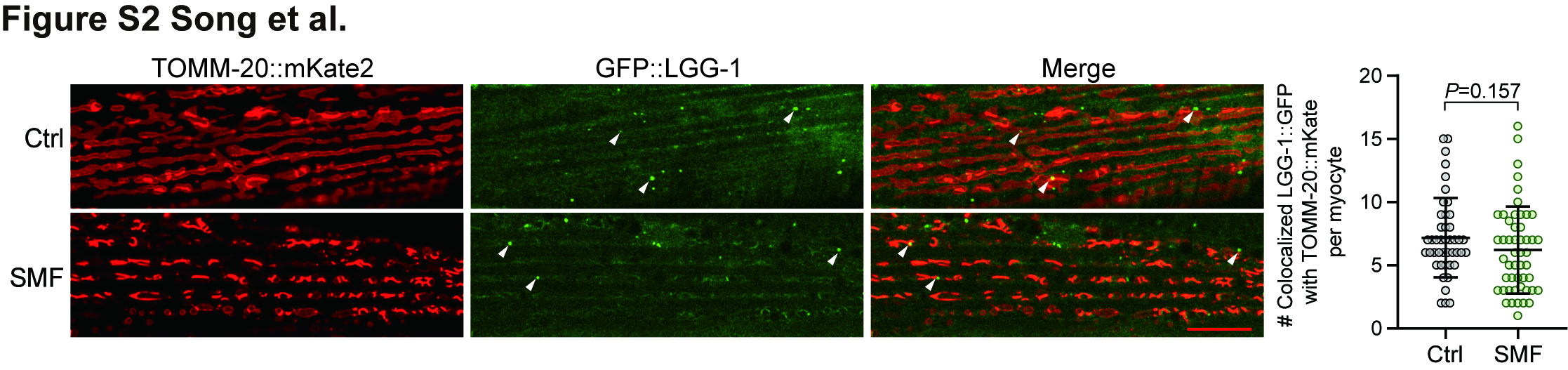

Supplement: Supplementary file 2 — Supplementary Figure S2. [file 41598_2022_20647_MOESM2_ESM.tif]

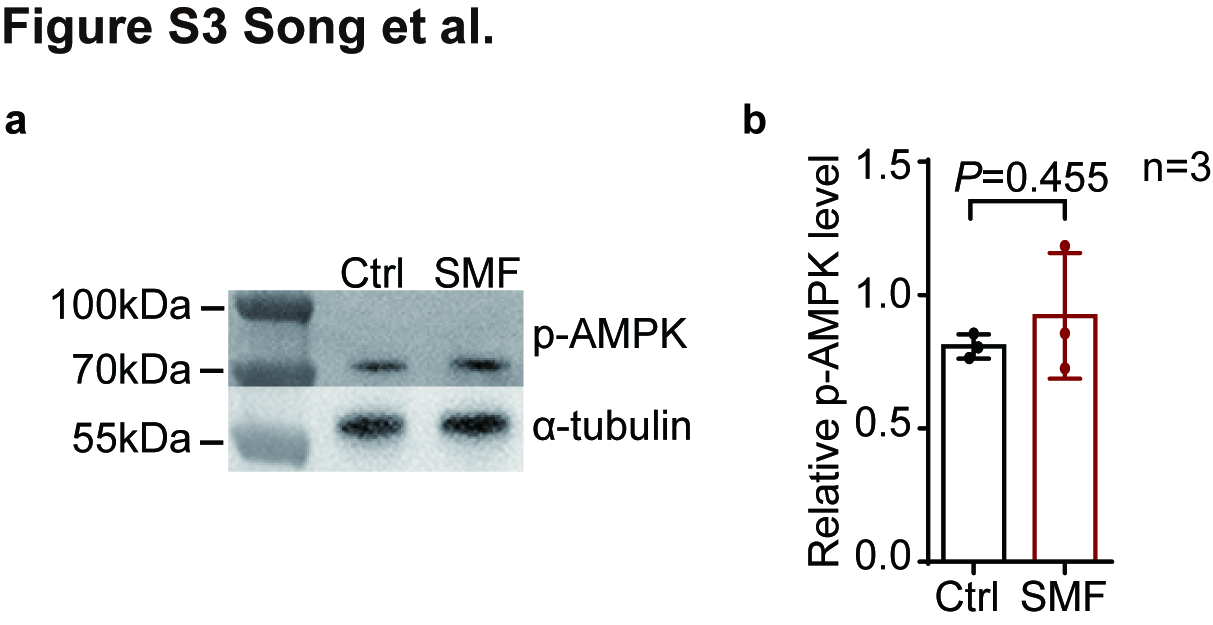

Supplement: Supplementary file 3 — Supplementary Figure S3. [file 41598_2022_20647_MOESM3_ESM.tif]

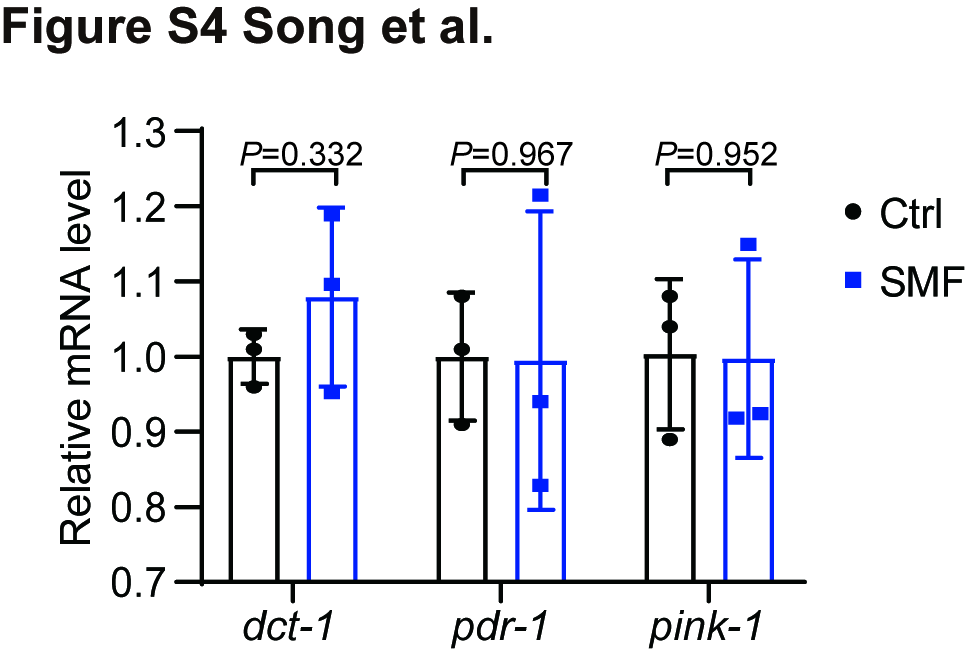

Supplement: Supplementary file 4 — Supplementary Figure S4. [file 41598_2022_20647_MOESM4_ESM.tif]

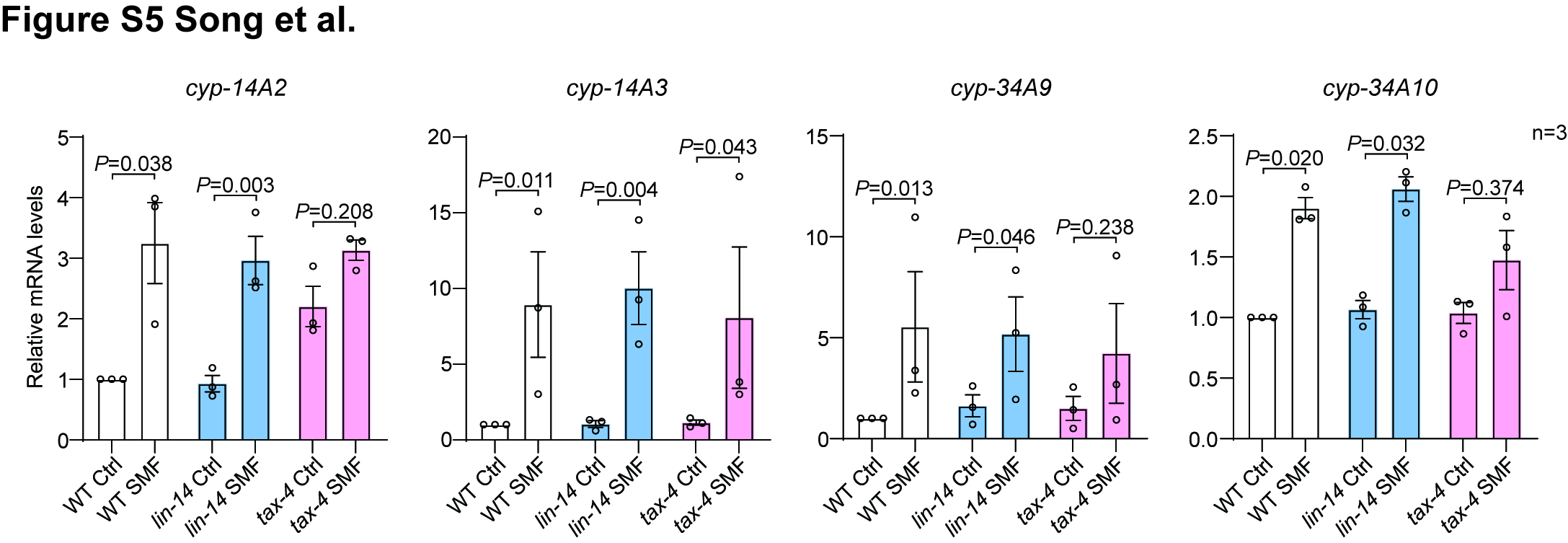

Supplement: Supplementary file 5 — Supplementary Figure S5. [file 41598_2022_20647_MOESM5_ESM.tif]

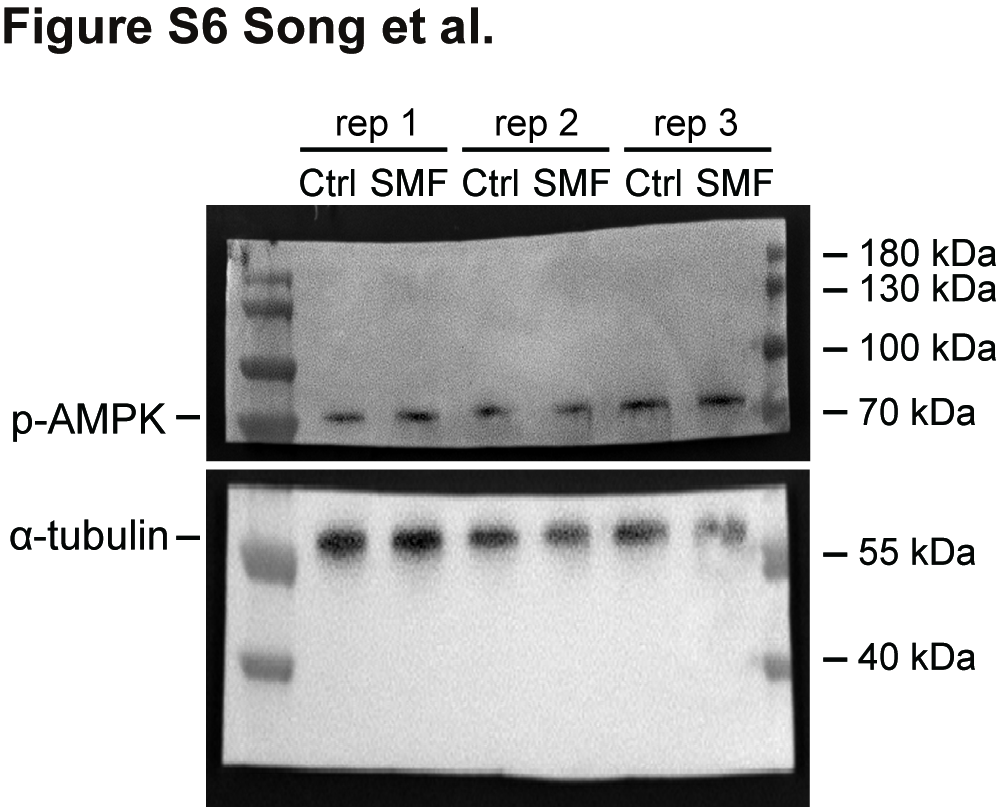

Supplement: Supplementary file 6 — Supplementary Figure S6. [file 41598_2022_20647_MOESM6_ESM.tif]
