# Supplementary material for: A moderate static magnetic field promotes C. elegans longevity through cytochrome P450s
Source: Sci Rep. 2022 Sep 27;12:16108. doi: 10.1038/s41598-022-20647-0 (PMC9515093; doi:10.1038/s41598-022-20647-0)
Supplement: Supplementary file 7 — Supplementary Legends. [file 41598_2022_20647_MOESM7_ESM.docx]

**A moderate static magnetic field promotes *C. elegans* longevity through cytochrome P450s**

**Authors:** Mengjiao Song^1^, Shiming Dong^1^, Xiangfei Zhang^2,3^, Yumin Dai^1^, Xin Zhang^2,3^, and Yidong Shen^1*^

**Affiliations:**

^1^ State Key Laboratory of Cell Biology, Innovation Center for Cell Signaling Network, CAS Center for Excellence in Molecular Cell Science, Shanghai Institute of Biochemistry and Cell Biology, University of Chinese Academy of Sciences， Chinese Academy of Sciences

320 Yueyang Rd.

200031 Shanghai, China

^2^ High Magnetic Field Laboratory, Hefei Institutes of Physical Science, Chinese Academy of Sciences

Hefei, China.

^3^ Science Island Branch of Graduate School, University of Science and Technology of China

Hefei, China.

*Correspondence to: yidong.shen@sibcb.ac.cn.

Tel: +86-21-54921171

**Supplementary information**

**Supplementary information contains 3 figures and 5 tables.**

**Supplementary Fig S1. A moderate static magnetic field improves the motility of aged worms.**

**(a)** The intensities of the SMFs.

**(b)** Worms were grown with or without SMF till indicated ages and measured for their thrashing rate at day 3 or day 10 of adulthood. Welch's *t*-test.

**Supplementary Fig S2. 10 mT SMF on mitophagy.**

The induction of mitophagy is indicated by co-localisation of GFP::LGG-1 (autophagosomes) and TOMM-20::mKate (mitochondria). The body wall muscle from the worms at day 10 of adulthood was examined. Scale bar: 10 μm. Unpaired *t*-test.

**Supplementary Fig S3. SMF of 10 mT does not alter AMPK activity.**

**(a) and (b)** Western blot (a) and corresponding quantification (b) of p-AMPK levels in D1 worms treated with or without 10 mT SMF. α-tubulin serves as a loading control for normalisation. Unpaired *t*-test.

**Supplementary Fig S4. The expression of mitophagy genes upon SMF treatment.**

RT-qPCR of the indicated genes in untreated worms (Ctrl) and worms grown in 10 mT SMF (SMF) at day 1 of adulthood. Unpaired *t*-test.

**Supplementary Fig S5. The expression of CYP genes in the mutants of *lin-14* or *tax-4* with or without SMF treatment.**

RT-qPCR of the indicated CYP genes in WT worms, *lin-14* mutants, and *tax-4* mutants at day 1 of adulthood. Worms were either untreated (Ctrl) or grown in 10 mT SMF (SMF). Unpaired *t*-test.

**Supplementary Fig S6. The full-length blots.**

The corresponding uncropped blots of Supplementary Fig S2a. The first biological replicate is shown in Supplementary Fig S2a. The quantitative results in Supplementary Fig S2b are from all three biological replicates.

**Supplementary Table 1. Detailed statistics of the assays in this study.**

**Supplementary Table 2. Differentially expressed genes by 10 mT SMF treatment**

**Supplementary Table 3. Gene set enrichment in the SMF-induced differentially expressed genes.**

**Supplementary Table 4. *C. elegans* strains used in this study.**

**Supplementary Table 5. Oligo sequences in this study.**
